# Mapping evolutionary paradigm of bovine viral diarrhea virus *Npro* associated with different organizations of nucleotide

**DOI:** 10.1080/21505594.2025.2550620

**Published:** 2025-08-29

**Authors:** Xili Feng, Zeyu Liu, Xiaoting Ren, Lele An, Xiao-Xia Ma

**Affiliations:** a Key Laboratory of Biotechnology and Bioengineering of State Ethnic Affairs Commission, Biomedical Research Center, Northwest Minzu University, Lanzhou, China; b Key Laboratory of Special Animal Epidemic Disease, Ministry of Agriculture, Institute of Special Animal and Plant Sciences, Chinese Academy of Agricultural Sciences, Changchun, China

**Keywords:** Bovine viral diarrhea virus, *Npro*, nucleotide pair, context-dependent codon bias, evolutionary paradigm

## Abstract

The non-structural protein (Npro) of bovine viral diarrhea virus (BVDV) is a crucial virulence factor that impairs the host’s antiviral immune response and facilitates virus production. This study establishes a foundation for understanding how different selective pressures influence the formation of nucleotide pairs, synonymous codon, and context-dependent codon bias (CDCB) in BVDV *Npro*. BVDV genotype 1 exhibits a greater number of subgenotypes compared to other genotypes, yet its overall nucleotide usage bias in *Npro* is stronger. Within *Npro*, certain dinucleotides, specifically CpG and UpA, are notably suppressed, while UpG is selected with high frequency across all genotypes. The BVDV *Npro* region exhibits a pronounced bias in synonymous codon usage and possesses a genetic capacity to distinguish between genotypes. Unlike the patterns of mononucleotide and synonymous codon usage associated with BVDV genotyping, nucleotide pair usage and CDCB show significant variability due to the high mutation rate in the Npro coding sequence. Despite this variation, both nucleotide architectures demonstrate a unique evolutionary paradigm that goes beyond genotype-specific models. Aside from nucleotide composition constraints imposed by the high mutation rate in the viral genome, natural selective pressures arising from translational selection and host immune response also significantly influence the formation of various nucleotide architectures in the BVDV *Npro*. By analyzing the genetic characterizations associated with the different nucleotide architectures in the *Npro*, the diverse repertoire of nucleotide pairs, synonymous codons and CDCB may provide BVDV mutants with ample opportunities for direct adaptation and exaptation, thereby overcoming the robust immune defenses of the host.

## Introduction

Bovine viral diarrhea virus (BVDV), a member of the *Pestivirus* genus within the *Flaviviridae* family, is the most prevalent infectious disease affecting cattle. Based on genetic differences, BVDV isolates have been classified into three genotype groups: BVDV-1 (genotype 1), BVDV-2 (genotype 2), and Hobi-like pestivirus (genotype 3), each exhibiting high genetic diversity and complex transmission cycles [1]. Notably, BVDV infection poses a significant threat to the cattle industry, as it can induce both reproductive and immunosuppressive effects resulting from either acute or persistent infection [2]. The clinical outcomes following infection with BVDV are complex and influenced by multiple factors, including the characteristics of the virus (notably the variation in virulence among different BVDV isolates), host factors (such as whether the host is immunotolerant or immunocompetent to BVDV), and environmental considerations (including concurrent infections with other pathogens) [3]. Most BVDV isolates exhibit low virulence and are associated with subclinical or very mild disease [4]. BVDV genotypes can be categorized into two biotypes: cytopathic (cp) and non-cytopathic (ncp). While cp BVDV strains are relatively rare and are primarily implicated in outbreaks of mucosal disease, ncp BVDV strains are more commonly encountered in nature and are frequently associated with the most severe forms of acute infection [2,5]. Furthermore, when susceptible pregnant cows are infected with ncp BVDV strains before their fetuses develop immunocompetence, this can result in the birth of PI calves [2,6].

The BVDV genome, composed of single-stranded, positive-sense RNA, encodes a polyprotein consisting of four structural proteins (C, Erns, E1 and E2) and eight non-structural proteins (Npro, p7, NS2, NS3, NS4A, NS4B, NS5A and NS5B) [7]. The viral protein products of BVDV play essential roles in various aspects of the virus’s lifecycle. The capsid protein is involved in the assembly with viral genomic RNA, while the envelope glycoproteins (E0, E1 and E2) contribute to the formation of lipid bilayers that encapsulate the virus particles. Non-structural proteins are critical in the processes of viral replication, transcription and translation, functioning both individually and cooperatively [2,8]. Infection with both cp and ncp BVDV strains triggers an inflammatory response and stimulates autophagy, which may impair the innate immune response in bovine cells and facilitate BVDV replication [9,10]. Additionally, BVDV infection is known to induce alterations in the host’s metabolic network; specifically, the downregulation of antiviral proteins and genes related to the complement system may contribute to enhanced viral proliferation [11]. The identification of long non-coding RNAs has provided fresh insights into gene regulation in the contexts of host-BVDV interactions [12]. Among the aforementioned viral proteins, Npro, which is encoded by a 504-nt coding sequence located at the 5’ terminus of the open reading frame (ORF) of BVDV, functions as a self-protease, facilitating the autocatalytic cleavage of the nascent BVDV polyprotein [13]. Notably, the *Pestivirus* genome is unique among the three genera of the *Flaviviridae* family, as it produces Npro, a protein not found in the other genera within this family [14]. BVDV Npro is a hydrophilic outer membrane protein characterized by a secondary structure rich in β-sheets and random coil regions; this protease possesses two active sites (Cys69 and His130) [15].

Viral host-cycling necessitates adaptation to the specific cellular environments and immune systems of diverse host species to perpetuate offspring [16–18]. All mammalian species rely on interferon (IFN) as a downstream effector, which is stimulated through pattern recognition receptors by pathogen-associated molecular patterns, in conjunction with B and T cell mediated adaptive immunity [19–22]. In both acute and persistent BVDV infections, Npro is capable of inhibiting type I interferon production by inducing the ubiquitination of IRF3 and interacting with multiple cellular factors [23–26]. These studies point out that BVDV Npro assists the virus into evading the innate antiviral response, thereby facilitating its viral lifecycle. In biological systems, the proper functioning of proteins significantly depends on their correct spatial folding during co-translation [27,28]. As viral protein products are crucial in interacting with and disrupting various host factors, most research on selective pressures has concentrated on the relationships between viral proteins and host cellular machinery. Furthermore, the nucleotide contexts within coding sequences play significant roles in regulating the folding processes of nascent polypeptide chains during co-translation [29–32]. A comprehensive investigation into evolutionary paradigm of the BVDV Npro coding sequence may give new genetic insights into the interactions between the viral life cycle and the host’s antiviral system. In this study, we conducted analyses of the evolutionary paradigm of Npro coding sequences derived from various BVDV strains across the three genotypes. The different organizations of nucleotide context regarding mononucleotide bias, dinucleotide bias, synonymous codon bias and context-dependent codon bias (CDCB) were calculated and analyzed by principal component analysis (PCA) for clarifying evolutionary paradigm of BVDV *Npro* region.

## Materials and methods

### Phylogenetic tree for BVDV Npro coding sequences

The advent of advanced sequencing technique for viral genomes has led to the cataloging of numerous BVDV genomes in the GenBank database at the National Center for Biotechnology Information (https://www.ncbi.nlm.nih.gov/genbank/). BVDV strains isolated within the same time frame and geographical region likely exhibit a high degree of genomic similarity and share genetic evolutionary relationships, primarily due to the spatiotemporal factors that limit genetic divergence during viral replication and transmission. In this study, we selected and downloaded 95 BVDV genomes, each verified as distinct genotypes and isolated from varied time periods and geographic locations (Table S1). The *Npro* region with 504-nt in length, was extracted from the corresponding BVDV ORF using multiple sequence alignments conducted with Clustal W (1.7). The *Pestivirus* genus encompasses BVDV, border disease virus (BDV) and classical swine fever virus (CSFV). These viruses exhibit cross-reactivity in serological assays and share similarities in protein structure, antigenic profiles, and genetic characteristics [33]. In this study, we focus primarily on BVDV, derived from bovine sources, while also incorporating a control group designed to facilitate cross-species comparisons within the *Pestivirus* genus. To clarify the genetic diversity of the *Npro* regions, we selected other *Pestivirus* members CSFV, NC_002657.1 and BDV, NC_003679.1) to serve as outgroup reference sequences. Phylogenetic analysis for the *Npro* regions was conducted using the neighbor-joining method within MEGA7.0 software. In the phylogenetic tree, the evolutionary distances were computed using the Maximum Composite Likelihood model with 1000 bootstrap replications.

### Estimation for usage bias of mononucleotide composition of BVDV Npro

First, the four mononucleotide compositions (U%, C%, A%, and G%) were calculated for both the entire coding sequence and individual codon positions. Based on the quantified mononucleotide composition data, Shannon entropy (information entropy) was employed to quantify the degree of nucleotide usage bias arising from the variations in the four mononucleotide compositions.

$$H=-\underset{x\in N}{\sum }p\left(x\right)\log\;p\left(x\right)$$

where Shannon entropy (*H*) is composed of the four mononucleotide composition frequencies, namely cytosine composition (C), guanine composition (G), uridine composition (U), and adenine composition (A). The “*N*” stands for the four mononucleotides. The *p*(*x*) stands for the probability of the specific mononucleotide in the target sequence.

### Estimation for nucleotide pair bias of BVDV Npro

For estimating nucleotide pair bias, the dinucleotide odds ratio is a widely used metric, specifically defined as *f*(*xy*)=*f*_*xy*_/*f*_*x*_*f*_*y*_. In this formula, *f*_*xy*_ denotes the frequency of a specific nucleotide pair (xy), while *f*_*x*_ and *f*_*y*_ represent the frequencies of nucleotides *x* and *y*, respectively. The dinucleotide odds ratio effectively highlights the discrepancies between the observed frequencies of nucleotide pairs and those expected based on the individual mononucleotide frequencies. Notably, a dinucleotide odds ratio of less than 0.78 indicates under-representation of the corresponding dinucleotide, while a value more than 1.23 signifies over-representation [34]. When the odds ratio falls within the range of 0.78 to 1.23, the associated nucleotide pair can be considered to exhibit unbiased dinucleotide usage in the respective sequence.

### Calculation for relative synonymous codon usage values of BVDV Npro

Based on the genetic phenomenon associated with synonymous codon usage bias in coding sequences, the relative synonymous codon usage value (RSCU) is widely recognized as a common and effective indicator for assessing the extent of usage bias among the target synonymous codons [35–37]. As for RSCU formula mentioned below, *A*_*ij*_ is the number of occurrences of the *j*^*th*^ codon for the *i*^*th*^ amino acid, which is encoded by *b*_*i*_ synonymous codons. Additionally, the *b* is the number of synonymous codons coding for the *i*^*th*^ amino acid.

$$\mathrm{RSCU}=\frac{A_{\mathrm{ij}}}{\sum_{j=1}^{b_{i}}A_{\mathrm{ij}}}\times b$$

Depending on CodonW software, we applied the RSCU formula to assess the degrees of usage bias associated with 59 synonymous codons of the BVDV *Npro* region. Due to high mutation rates in the BVDV genome [38,39], the RSCU values for the *Npro* region were estimated based on the mean value and the 95% confidence interval. According to RSCU data for the Npro coding sequences analyzed in this study, the extent of synonymous codon usage bias can be assessed by the standard established in previous studies, namely overrepresented synonymous codons with RSCU > 1.6 and underrepresented codons with RSCU < 0.6 [40,41].

### Assessment for natural selective pressure influencing the overall codon usage of BVDV Npro

In contrast to the RSCU value, which only reflects the usage bias of a single synonymous codon, the effective number of codons (ENC) is commonly used to evaluate the overall codon usage bias of the target whole coding sequence [42,43]. We assessed the relationships between ENC and GC3 content (the composition of guanine plus cytosine at the third codon position) in the BVDV *Npro* region. Additionally, a linear regression method was employed to estimate the role of compositional constraint associated with GC3 content in the overall codon usage bias of the *Npro* region. Furthermore, the plot composed of ENC vs. GC3 content was constructed by GraphPad Prism software.

### Assessment for context-dependent codon bias of BVDV Npro

Mononucleotide, nucleotide pair, and synonymous codons can be selected nonrandomly in coding sequences, while neighboring nucleotides surrounding a codon (a specific form of tetranucleotide) influence the selection of that codon from the synonymous family. This genetic phenomenon is referred to as context-dependent codon bias (CDCB) [44,45]. Moreover, the most important nucleotide shaping CDCB is the first nucleotide immediately following the codon and is referred to as the N_1_ context [46–48]. After estimating usage biases for mononucleotides, nucleotide pairs, and codons in the BVDV *Npro* region, we further analyzed CDCB of the BVDV *Npro* region. For quantifying the CDCB of Npro *Npro* region, we introduced the context N_1_, referred to as XYZ_N (where X, Y, Z and N represent any nucleotide), which indicates the nucleotide immediately following the codon in the computation of the *R* value reflecting the strength of CDCB in *Npro* region. Here, the *R* value is defined as the relative abundance of a codon (XYZ) with N calculated as the ratio R_(XYZ_N)_ = F_(XYZ_N)_/F_(XYZ)_F_(N)_. In this formula, F_(XYZ_N)_ represents the frequency of the codon in the N_1_ context, F_(XYZ)_ denotes the frequency of the codon (XYZ), and F_(N)_ represents the frequency of nucleotide N in the N_1_ context.

### Principal component analyses for different nucleotide organizations

Based on data related to mononucleotide, nucleotide pairs, synonymous codons, and CDCB of the BVDV *Npro* region, we employed PCA to visualize evolutionary patterns related to the four nucleotide organizations. The PCA method is a widely used technique for summarizing the distance matrix, recording the distances between each pair of data samples [49,50]. Here, we employed the PCA method to visualize evolutionary patterns associated with information entropy, dinucleotide odds ratio, RSCU, and *R* value, respectively. When PCA was conducted to analyze the overall nucleotide usage bias, the data matrix comprised three variables related to the information entropy of the three codon positions in the *Npro* region. When PCA was performed to estimate nucleotide pair bias, the data matrix comprised 16 variables related to the dinucleotide odds ratio across the entire coding sequence or within different codon positions of Npro. When PCA was utilized to visualize synonymous codon usage bias, the data matrix comprised 59 variables associated with RSCU in the *Npro* region. Additionally, when PCA was applied to visualize the evolutionary pattern derived from CDCB, the data matrix comprised 258 variables related to the *R* value in the *Npro* region. Depending on the scatterplot3d package, the 3D map visualizing the evolutionary patterns associated with the specific nucleotide organization of *Npro* region is formed by the first three principal components of the PCA method.

### Statistical analyses

The data involved in our study were analyzed using a One-way ANOVA, which assesses the influence of a single independent variable on the analysis of variance. In One-way ANOVA analyses, the least significant difference (LSD) method, which is based on *t* tests for pariwise comparisons between group averages, was utilized for post-hoc testing using SPSS 16.0 for Windows. A significant difference can be identified when *p* value was < 0.05.

## Results

### BVDV Npro region owns the genotype-specific model

Since these BVDV *Npro* regions analyzed here have been identified for genotyping classification through phylogenetic analysis of the BVDV 5’UTR regions, we further investigated whether the *Npro* region possess the ability to classify genotypes. In contrast to the two outgroup *Pestivirus* members (CSFV and BDV), the *Npro* regions of BVDV exhibited a genotype-specific model (Figure S1). In comparison to the evolutionary distance between genotype 1 and genotype 2 regarding the *Npro* regions, genotype 3 displayed a substantially divergent evolutionary clade (Figure S1). Although genotype 1 and genotype 2 of the *Npro* region exhibited the relatively close evolutionary distances, the two genotypes constituted sister evolutionary clades, with the *Npro* regions within genotype 1 displaying high genetic diversity (Figure S1).

### Significant variable of nucleotide usage bias of the Npro region within different genotypes

**Figure 1.**
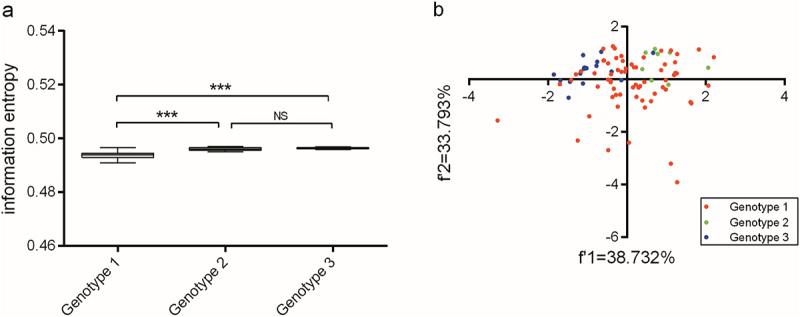
The overall nucleotide usage patterns among BVDV Npro coding sequences.

Before estimating for nucleotide usage bias of the *Npro* region, we first clarified the mononucleotide composition in both the complete coding sequence and specific codon positions. In the three genotypes, the A+U composition ( >50%) was greater than the G+C composition in both the three codon positions and the entire coding sequence of BVDV Npro (Table S2). Based on the calculated variations in mononucleotide composition for both the coding sequence and specific codon positions of BVDV Npro, we subsequently quantified the overall nucleotide usage bias for the entire coding sequence. The overall nucleotide usage bias of the genotype 1 *Npro* was significantly stronger (*p* < 0.001), while the overall nucleotide usage bias of the genotype 2 *Npro* was similar to that of genotype 3 (*p* > 0.05) (Figure 1A), suggesting that both genotype 2 and genotype 3 own the more similar mononucleotide composition skew than that of genotype 1. Moreover, depending on PCA, the nucleotide usage biases in the three codon positions of BVDV *Npro* did not exhibit a genotype-specific model, and the nucleotide usage variations in the three codon positions of genotype 1 *Npro* were more variability than those of the other two genotypes (Figure 1B).

(a) The overall codon usage bias of Npro with the certain genotype represented by information entropy. “***” means the *P* value < 0.001 and “NS” means the *P* value > 0.05. (b) the overall nucleotide usage pattern of Npro with the certain genotype illustrated by PCA method. The two factors (f’1 and f’2) are calculated by PCA method.

### BVDV Npro owning substantial variation in nucleotide pair usage

**Figure 2.**
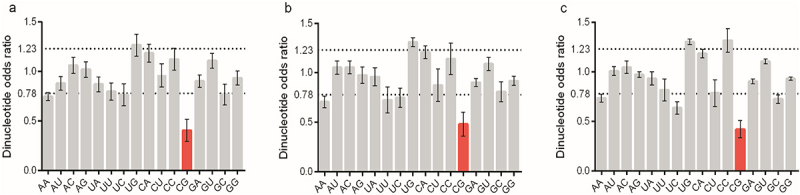
The nucleotide pair bias for BVDV Npro coding sequence represented by dinucleotide odds ratio. (a) 16 nucleotide pairs for Npro with genotype 1. (b) 16 nucleotide pairs for Npro with genotype 2. (c) 16 nucleotide pairs for Npro with genotype 3.

The dinucleotide odds ratios for the 16 dinucleotides were initially compared across the entire coding sequence of Npro. Previous reports indicated that dinucleotide odds ratios >1.23 are considered indicative of overrepresented nucleotide pairs, whereas values < 0.78 suggest underrepresented nucleotide pairs [51]. As shown in Figure 2, a schematic depiction regarding the dinucleotide odds ratios was made for the *Npro* regions of each BVDV genotype. The nucleotide pairs ApU, ApC, ApG, UpA, GpA, GpC, GpU, and GpG, showed no overall bias in any of the analyzed BVDV genotypes, as their dinucleotide odds ratios fell within the normal range for at least 50% of the component data sets. In contrast, the nucleotide pair ApA and CpG were significantly underrepresented, whereas the dinucleotide UpG was significantly overrepresented across the analyzed BVDV genotypes.

**Figure 3.**
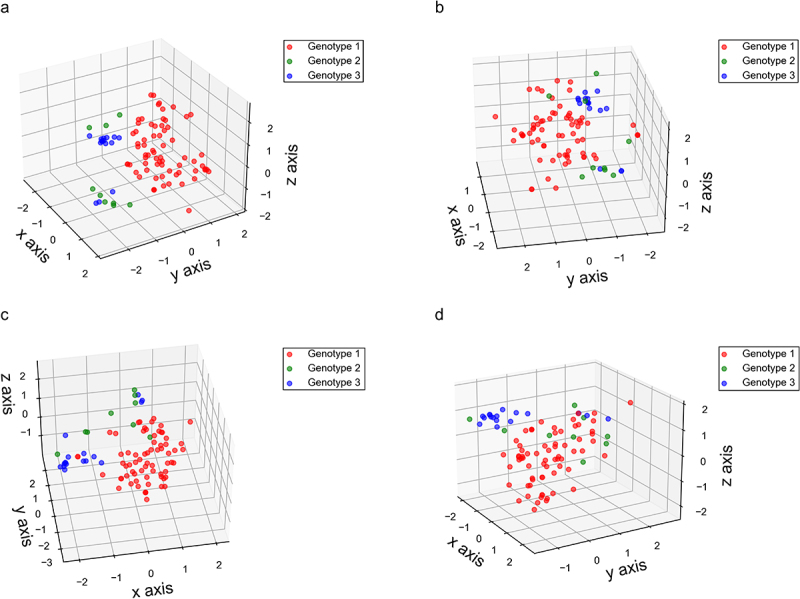
Evolutionary paradigm regarding nucleotide pair usage in BVDV Npro coding sequence illustrated by PCA method. (a) the whole coding sequence of BVDV Npro. The ×axis, y axis, and z axis are *f’*_*1*_ = 16.329%, *f’*_*2*_ = 14.321%, and *f’*_*3*_ = 10.741, respectively. (b) the first and second positions in BVDV Npro coding sequence. The ×axis, y axis, and z axis are *f’*_*1*_ = 29.002%, *f’*_*2*_ = 15.196%, and *f’*_*3*_ = 14.024%, respectively. (c) the second and third positions in BVDV Npro coding sequence. The ×axis, y axis, and z axis are *f’*_*1*_ = 25.689%, *f’*_*2*_ = 23.081%, and *f’*_*3*_ = 13.823%, respectively. (d) the third position of a code and the first position of the downstream code in BVDV Npro coding sequence. The ×axis, y axis, and z axis are *f’*_*1*_ = 19.077%, *f’*_*2*_ = 18.754%, *f’*_*3*_ = 13.510%, respectively.

PCA was employed to visualize the evolutionary patterns of nucleotide pair usage in the BVDV *Npro* region. Although the nucleotide pair usage patterns of the different genotypes varied considerably, the nucleotide pair usage pattern of genotype 1 *Npro* generally separated from those of genotypes 2 and 3; whereas the nucleotide pair usage patterns of genotypes 2 and 3 exhibited a mixture and clustered into two distinct groups (Figure 3A). Furthermore, PCA was also employed to visualize the evolutionary patterns associated with nucleotide pair usage at different codon positions (the first and second codon positions, the second and third codon positions, and the third position of a codon and first codon position of a downstream codon) of BVDV *Npro* region. Similar evolutionary patterns regarding nucleotide pair usage were observed in both the first and second codon positions as well as the second and third codon positions (Figure 3B,C). For nucleotide pair usage at the third position of a codon and the first position of a downstream codon, the evolutionary patterns also displayed a clear separation of genotypes 2 and 3 from genotype 1 (Figure 3D). Although the evolutionary patterns related to nucleotide pair usage in the BVDV *Npro* region did not demonstrate a genotype-specific model, the genetic characteristics associated with nucleotide pair usage indicate that these pairs may capture additive evolutionary information pertinent to the BVDV *Npro* region.

### BVDV Npro owning genotype-specific model in synonymous codon usage

After analyzing genetic features regarding the overall nucleotide usage and nucleotide pair usage, we further investigated synonymous codon usage in BVDV Npro coding sequence through RSCU calculation. According to data of mean value with 95% confidence interval for BVDV Npro coding sequence, the amount of underrepresented synonymous codon was higher than that of overrepresented one in the three genotypes. In genotype 1, the following overrepresented synonymous codons were identified: AGU for Ser, ACA for Thr, and AGA for Arg; the underrepresented codons included UCC and UCG for Ser, CCG for Pro, ACG for Thr, GCG for Ala, CGU, CGC, CGA and CGG for Arg, and GGC for Gly (Table 1). In genotype 2, the overrepresented codons contained AUC for Ile, AGU and AGC for Ser, CCA for Pro, AGA for Arg; the underrepresented codons were UUC for Phe, CUU for Leu, AUU and AUA for Ile, UCU and UCG for Ser, CCC for Pro, ACG for Thr, AAG for Lys, CGU and CGC for Arg (Table 2). In genotype 3, the overrepresented codons were AGU for Ser, ACA for Thr, GCC for Ala, AGA and AGG for Arg; the underrepresented ones owned CUA for Leu, AUU for Ile, UCG for Ser, CCU for Pro, ACU and ACG for Thr, GCG for Ala, CAU for His, GAC for Asp, CGA and CGG for Arg (Table 3). Notably, most synonymous codons containing the CpG dinucleotide were significantly suppressed in their usage. Moreover, the three genotypes were able to share the two overrepresented codons (AGU for Ser and AGA for Arg) and the two underrepresented ones (UCG for Ser and ACG for Thr). These results indicate that strong selective pressures impose limitations on changes in synonymous codon usage within the BVDV Npro coding sequence.

**Table 1.** Synonymous codon usage variance for genotype 1.

Synonymous codon (amino acid) in Npro coding sequence	The average RSCU value	95% confidence interval	
		Lower bond	Upper bond
UUU(F)	1.44	1.34	1.54
UUC(F)	0.56	0.46	0.66
UUA(L)	1.23	1.09	1.36
UUG(L)	1.60	1.47	1.72
CUU(L)	0.63	0.57	0.70
CUC(L)	0.68	0.58	0.78
CUA(L)	1.01	0.89	1.12
CUG(L)	0.85	0.73	0.97
AUU(I)	0.91	0.81	1.00
AUC(I)	0.85	0.76	0.94
AUA(I)	1.24	1.13	1.36
GUU(V)	0.67	0.57	0.77
GUC(V)	1.05	0.96	1.14
GUA(V)	0.89	0.80	0.98
GUG(V)	1.39	1.31	1.47
UCU(S)	0.47	0.34	0.61
UCC(S)	0.38	0.27	0.48
UCA(S)	1.13	1.00	1.26
UCG(S)	0.47	0.36	0.57
**AGU(S)**	1.90	1.75	2.05
AGC(S)	1.66	1.49	1.82
CCU(P)	0.82	0.72	0.93
CCC(P)	1.03	0.93	1.13
CCA(P)	1.66	1.57	1.76
CCG(P)	0.49	0.41	0.57
ACU(T)	0.90	0.76	1.04
ACC(T)	0.67	0.56	0.78
**ACA(T)**	2.01	1.88	2.14
ACG(T)	0.42	0.35	0.49
GCU(A)	1.07	0.87	1.27
GCC(A)	1.24	1.09	1.38
GCA(A)	1.32	1.11	1.53
GCG(A)	0.38	0.25	0.50
UAU(Y)	0.73	0.67	0.79
UAC(Y)	1.27	1.21	1.33
CAU(H)	0.60	0.49	0.71
CAC(H)	1.40	1.29	1.51
CAA(Q)	1.25	1.16	1.34
CAG(Q)	0.75	0.66	0.84
AAU(N)	1.11	1.01	1.21
AAC(N)	0.89	0.79	0.99
AAA(K)	1.27	1.20	1.35
AAG(K)	0.73	0.65	0.80
GAU(D)	1.06	0.98	1.14
GAC(D)	0.94	0.86	1.02
GAA(E)	1.07	1.02	1.12
GAG(E)	0.93	0.88	0.98
UGU(C)	0.88	0.80	0.96
UGC(C)	1.12	1.04	1.20
CGU(R)	0.37	0.26	0.49
CGC(R)	0.19	0.12	0.26
CGA(R)	0.09	0.04	0.14
CGG(R)	0.19	0.12	0.26
**AGA(R)**	3.57	3.39	3.74
AGG(R)	1.59	1.38	1.80
GGU(G)	1.01	0.94	1.08
GGC(G)	0.49	0.41	0.57
GGA(G)	1.20	1.13	1.28
GGG(G)	1.30	1.24	1.36

As for verifying the underrepresented synonymous codon, the RSCU value which is determined by the upper bond less than 0.6; as for verifying the over-represented one, the value which is determined by the lower bond is more than 1.6. The synonymous codons with underline were defined as underrepresented codon; the synonymous codons with bond font were defined as overrepresented one.

**Table 2.** Synonymous codon usage variance for genotype 2.

Synonymous codon (amino acid) in Npro coding sequence	The average RSCU value	95% confidence interval	
		Lower bond	Upper bond
UUU(F)	1.67	1.12	2.21
UUC(F)	0.33	^a^-0.21	0.88
UUA(L)	1.78	1.50	2.06
UUG(L)	0.64	0.52	0.76
CUU(L)	0.40	0.30	0.50
CUC(L)	0.49	0.34	0.64
CUA(L)	1.32	1.06	1.58
CUG(L)	1.37	1.20	1.53
AUU(I)	0.25	0.05	0.45
**AUC(I)**	2.40	2.23	2.57
AUA(I)	0.36	0.32	0.39
GUU(V)	0.77	0.42	1.12
GUC(V)	1.39	1.00	1.78
GUA(V)	0.53	0.32	0.75
GUG(V)	1.31	1.17	1.45
UCU(S)	0.07	^a^-0.09	0.22
UCC(S)	1.15	0.99	1.31
UCA(S)	0.61	0.59	0.62
UCG(S)	0.00	^b^——	^b^——
**AGU(S)**	2.16	1.81	2.52
**AGC(S)**	2.01	1.63	2.40
CCU(P)	0.55	0.37	0.73
CCC(P)	0.37	0.21	0.52
**CCA(P)**	2.09	1.86	2.32
CCG(P)	0.99	0.82	1.16
ACU(T)	1.04	0.94	1.14
ACC(T)	1.43	1.28	1.58
ACA(T)	1.47	1.24	1.70
ACG(T)	0.06	^a^-0.07	0.18
GCU(A)	0.76	0.39	1.14
GCC(A)	0.85	0.54	1.16
GCA(A)	1.25	0.96	1.55
GCG(A)	1.13	0.77	1.50
UAU(Y)	0.84	0.67	1.01
UAC(Y)	1.16	0.99	1.33
CAU(H)	0.83	0.56	1.11
CAC(H)	1.17	0.89	1.44
CAA(Q)	1.37	1.14	1.60
CAG(Q)	0.63	0.40	0.86
AAU(N)	1.12	0.97	1.27
AAC(N)	0.88	0.73	1.03
AAA(K)	1.66	1.57	1.75
AAG(K)	0.34	0.25	0.43
GAU(D)	0.79	0.60	0.98
GAC(D)	1.21	1.02	1.40
GAA(E)	1.23	1.17	1.28
GAG(E)	0.77	0.72	0.83
UGU(C)	0.96	0.78	1.14
UGC(C)	1.04	0.86	1.22
CGU(R)	0.24	0.07	0.42
CGC(R)	0.00	^b^——	^b^——
CGA(R)	0.58	0.40	0.75
CGG(R)	0.77	0.62	0.92
**AGA(R)**	2.47	2.01	2.93
AGG(R)	1.95	1.56	2.33
GGU(G)	0.96	0.70	1.21
GGC(G)	0.98	0.71	1.25
GGA(G)	0.86	0.70	1.02
GGG(G)	1.20	1.02	1.38

^a^The value indicates that the selection frequency for the corresponding synonymous codon is so low that the codon is even not selected by genotype 2 Npro.

^b^The RSCU values for the synonymous codon UCG and CGC are constant, thus the associated calculations for 95% confidence interval are omitted.

As for verifying the underrepresented synonymous codon, the RSCU value which is determined by the upper bond less than 0.6; as for verifying the over-represented one, the value which is determined by the lower bond is more than 1.6. The synonymous codons with underline were defined as underrepresented codon; the synonymous codons with bond font were defined as overrepresented one.

**Table 3.** Synonymous codon usage variance for genotype 3.

Synonymous codon (amino acid) in Npro coding sequence	The average RSCU value	95% confidence interval	
		Lower bond	Upper bond
UUU(F)	1.44	1.29	1.58
UUC(F)	0.56	0.42	0.71
UUA(L)	1.66	1.54	1.77
UUG(L)	1.15	0.98	1.32
CUU(L)	0.53	0.39	0.66
CUC(L)	0.54	0.45	0.64
CUA(L)	0.42	0.24	0.60
CUG(L)	1.70	1.51	1.90
AUU(I)	0.38	0.20	0.56
AUC(I)	1.27	1.09	1.45
AUA(I)	1.35	1.23	1.47
GUU(V)	0.75	0.63	0.87
GUC(V)	0.63	0.51	0.76
GUA(V)	1.23	1.10	1.36
GUG(V)	1.38	1.25	1.52
UCU(S)	0.74	0.64	0.84
UCC(S)	0.72	0.63	0.82
UCA(S)	0.49	0.30	0.68
UCG(S)	0.04	^a^-0.05	0.14
**AGU(S)**	2.39	2.20	2.59
AGC(S)	1.61	1.39	1.83
CCU(P)	0.32	0.23	0.36
CCC(P)	1.41	1.37	1.46
CCA(P)	1.29	1.18	1.41
CCG(P)	0.97	0.85	1.10
ACU(T)	0.00	^b^—–	^b^——
ACC(T)	1.41	1.24	1.58
**ACA(T)**	2.44	2.27	2.61
ACG(T)	0.15	0.03	0.28
GCU(A)	0.70	0.47	0.93
**GCC(A)**	2.03	1.80	2.25
GCA(A)	1.23	1.09	1.37
GCG(A)	0.04	^a^-0.04	0.12
UAU(Y)	1.33	1.27	1.39
UAC(Y)	0.67	0.61	0.73
CAU(H)	0.47	0.39	0.55
CAC(H)	1.53	1.45	1.61
CAA(Q)	0.87	0.78	0.97
CAG(Q)	1.13	1.03	1.22
AAU(N)	1.07	0.98	1.16
AAC(N)	0.93	0.84	1.02
AAA(K)	1.18	1.13	1.23
AAG(K)	0.82	0.77	0.87
GAU(D)	1.53	1.46	1.59
GAC(D)	0.47	0.41	0.54
GAA(E)	1.08	1.00	1.16
GAG(E)	0.92	0.84	1.00
UGU(C)	0.86	0.76	0.96
UGC(C)	1.14	1.04	1.24
CGU(R)	0.57	0.41	0.74
CGC(R)	0.57	0.41	0.74
CGA(R)	0.07	^a^-0.03	0.16
CGG(R)	0.04	^a^-0.04	0.11
**AGA(R)**	2.13	1.97	2.29
**AGG(R)**	2.62	2.46	2.78
GGU(G)	0.61	0.52	0.69
GGC(G)	1.15	1.07	1.23
GGA(G)	0.97	0.90	1.05
GGG(G)	1.27	1.19	1.35

^a^The value indicates that the selection frequency for the corresponding synonymous codon is so low that the codon is even not selected by genotype 2 Npro.

^b^RSCU value for the synonymous codon ACU is constant. It has been omitted from the calculation of 95% confidence interval.

As for verifying the underrepresented synonymous codon, the RSCU value which is determined by the upper bond less than 0.6; as for verifying the over-represented one, the value which is determined by the lower bond is more than 1.6. The synonymous codons with underline were defined as underrepresented codon; the synonymous codons with bond font were defined as overrepresented one.

### Natural selection and mutation pressure influencing BVDV *Npro* evolution

**Figure 4.**
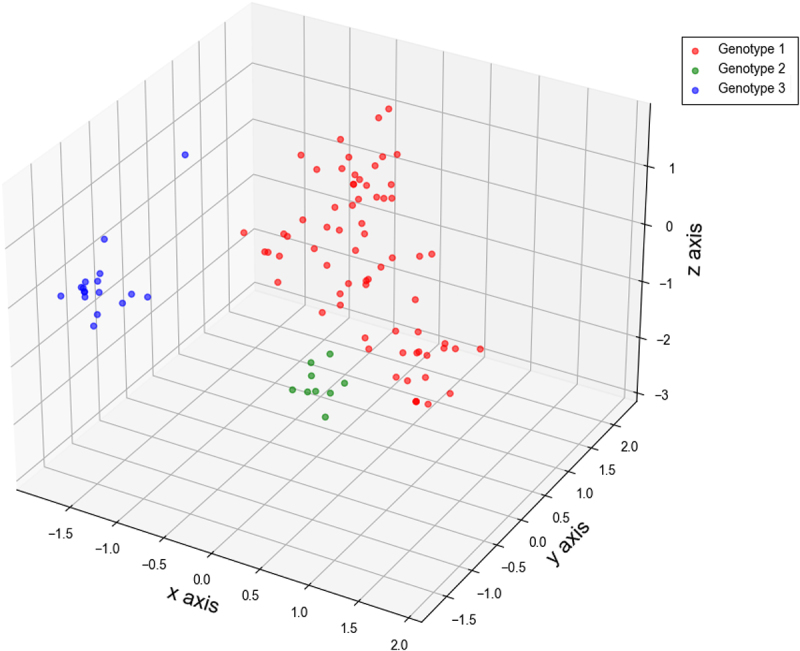
Evolutionary paradigm regarding synonymous codon usages for BVDV Npro coding sequence illustrated by PCA method. The ×axis, y axis, and z axis are *f’*_*1*_ = 14.766%, *f’*_*2*_ = 11.372%, *f’*_*3*_ = 9.747%, respectively.

**Figure 5.**
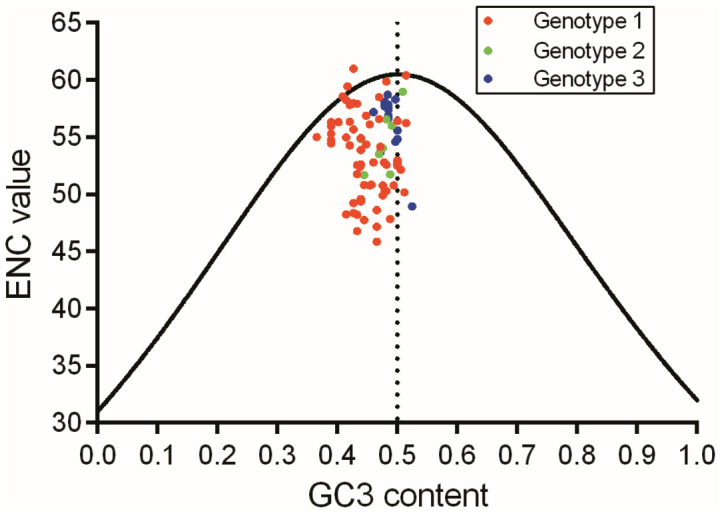
The plot of ENC vs. GC3 content for BVDV Npro coding sequence.

Based on the RSCU data of BVDV Npro, the PCA method was employed to visualize the evolutionary paradigm of the Npro coding sequence regarding synonymous codon usage. As shown in Figure 4, although the three genotypes exhibited a genotype-specific model of synonymous codon usage in Npro coding sequence, each genotype demonstrated highly variable synonymous codon usages, particular genotype 1. The evolutionary paradigm also reflected that the nucleotide composition constraint caused by mutation pressure remains one of the critical genetic factors for BVDV Npro evolution. To further validate the two primary factors (nucleotide composition constraint and natural selective pressure) in influencing the overall codon usage bias, the plot was made up of ENC vs. GC3 content raised from BVDV Npro coding sequence. As shown in Figure 5, the majority of dots fell below the expected trendline rather than aligning with it. Nevertheless, the extent of the overall codon usage bias varied considerably among the three genotypes. This genetic characteristic indicated that both nucleotide composition constraints and natural selective pressure play significant roles in the synonymous codon usage of BVDV Npro coding sequence.

### Discrepancies in context-dependent codon bias for BVDV *Npro*

**Figure 6.**
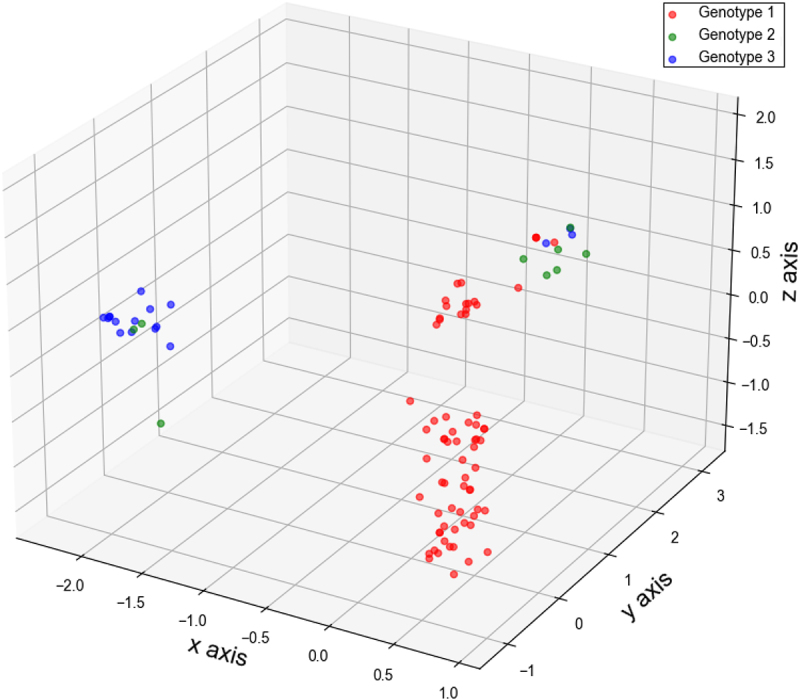
Evolutionary paradigm regarding CDCB for BVDV Npro coding sequence illustrated by PCA method. The ×axis, y axis, and z axis are *f’*_*1*_ = 17.619%, *f’*_*2*_ = 8.089%, *f’*_*3*_ = 7.382%, respectively.

Based on the aforementioned data regarding nucleotide usage bias, nucleotide pair bias, and synonymous codon usage bias for BVDV Npro, we further calculated *R* values to highlight CDCB for BVDV *Npro* in each genotype. The *R* values for each BVDV strain analyzed in this study exhibited considerable variability (Table S3), indicating the discrepancy in synonymous codon bias concerning adjacent nucleotide types in the BVDV Npro coding sequence. Furthermore, to assess the role of nucleotide composition constraints imposed by mononucleotide composition variants in the first codon position of *Npro*, we analyzed the CDCB associated with overrepresented synonymous codons in each genotype (Table 4). Based on mononucleotide composition variants in the first codon position of each genotype (Table S1), the overrepresented synonymous codons exhibiting favorable or unfavorable nucleotide compositions strongly indicated that nucleotide composition constraints from mononucleotide variants in the first codon position did not dominant the CDCB of overrepresented codons (Table 4). In addition, PCA was utilized to elucidate the evolutionary patterns of BVDV *Npro* with respect to the *R* values. Although the *R* values for CDCB in BVDV *Npro* had limited capacity to distinguish the three genotypes, a significant number of data points representing genotype 1 remained distinct from the other two genotypes, revealing three clearly observable evolutionary groups (Figure 6). The genetic characteristics concerning CDCB suggest that natural selective pressure plays a significant role in the evolutionary patterns of BVDV *Npro*.

**Table 4.** The overrepresented synonymous codon closely flanked by the favorable/unfavorable downstream nucleotide.

	Overrepresented synonymous codon with favorable neighboring nucleotide	Overrepresented synonymous codon with unfavorable neighboring nucleotide
Genotype 1	AGU_G	AGU_A
	ACA_A	ACA_C
	AGA_G	AGA_C
Genotype 2	AUC_T	AUC_G
	AGU_G	AGU_A
	AGC_C	AGC_T
	CCA_G	CCA_T
	AGA_G	AGA_C
Genotype 3	AGU_T	AGU_C
	ACA_G	ACA_T
	GCC_C	GCC_T
	AGA_G	AGA_T
	AGG_A	AGG_G

## Discussion

In the evolutionary trajectory of BVDV, a high frequency of mutations, a propensity for recombination, and selective pressures from immune responses triggered by natural infection have resulted in a diverse array of genetic and antigenic variants of the virus. Typically, the genetic variants of BVDV isolates can be classified through phylogenetic analysis. The most frequently utilized regions of the BVDV genome for genotyping are the 5’UTR and the *Npro* region, as these areas are more conserved compared to other regions (e.g. the *E2* region) [5,52–54]. As illustrated in Figure S1, the *Npro* region exhibits a genotypic-specific model that reflects the homology of aligned nucleic acid sequences. The IFN pathway, as a key component of innate immunity, serves as the first line of defense against viral infection in all animals [55]. Natural selective pressure arising from interferon antiviral actions can impose a strong limitation on viral progeny production, subsequently influencing the viral evolutionary pathway [56–59]. BVDV Npro can negatively regulate IFN responses, thereby weakening the host’s antiviral actions [25,26], resulting in increased viral progeny production and the emergence of mutation variants. Since Npro can diminish the natural selective pressure arising from the IFN antiviral response, this viral nonstructural protein may exhibit a unique evolutionary paradigm regarding nucleotide usage patterns. Although the mononucleotide composition in different codon positions of the *Npro* region varied considerably among the genotypes (Table S1 and Figure 1B), genotype 1 *Npro* exhibited a stronger bias in nucleotide usage compared to the other genotypes (Figure 1A). In genetic studies of BVDV isolates, the *Npro* region is commonly utilized for identifying subtype groups and demonstrates a novel performance in the identification of genotype 1 [54,60–63]. The overall nucleotide usage bias in the *Npro* region of genotype 1 facilitated the effective genotype classification. Nucleotide pair usage patterns (such as CpG and UpG) still indicated that natural selective pressures arising from immune response and translational limitations played significant roles in BVDV Npro (Figure 2), even though nucleotide pair usage at different codon positions exhibited limited power to clarify a genotype-specific model (Figure 3). This genetic phenomenon has been observed in the genomes of various organisms (including viruses and microorganisms) and is attributed to a direct consequence of dinucleotide bias [34,47,64–68]. Apart from factors related to DNA secondary structures and dinucleotide stacking energies, cytosine methylation is believed to play a crucial role in the suppression of dinucleotide CpG in vertebrates [51,69–71]. Moreover, the weak usage of dinucleotide UpA and the strong suppressive of dinucleotide CpG in BVDV Npro coding sequence suggested that translational selection from host cell regarding gene replication and immune response contributes significantly to the evolutionary paradigm of BVDV *Npro*. The suppression of dinucleotide CpG motifs can weaken antiviral defenses that target non-self RNA (e.g. zinc-finger antiviral protein), thereby impairing the innate immune response against viral infections [72–75]. Furthermore, the frequencies of dinucleotide CpG and UpA significantly regulate RNA virus replication ability and translation efficiency [76–78].

Nucleotide and codon usage biases are frequently influenced by factors related to species or protein functions [79]. Codon usage bias, characterized by the preferential use of certain codons over their synonymous counterparts, is an intriguing phenomenon influenced by three selective forces: mutation pressure, natural selective pressure, and genetic drift. Despite high mutation rates in virus’s genome in particular RNA viruses RNA viruses, the pronounced usage bias of synonymous codons can serve as a significant genetic characteristic in the evolution of virus-host interactions [36,80–82]. According to the synonymous codon usage patterns of BVDV *Npro* (Tables 1-3; Figures 4 and 5), the results revealed that this codon usage bias is driven primarily by two factors: natural selective pressure from the host and nucleotide composition constraints arising from viral mutation pressure. Moreover, as illustrated in Figure 5, the majority of BVDV *Npro* regions are influenced by natural selective pressures that contribute to a pronounced overall codon usage bias. These selective pressures encompass various factors, including host proteins that recognize specific nucleic acid patterns. When these patterns are detected, they can trigger mutations or activate intrinsic and innate immune responses, further shaping the viral genomic landscape [83–88]. Generally, translational selection or codon usage bias is more frequently observed in unicellular organisms (including viruses) compared to eukaryotes, where a favorable subset of synonymous codons is closely associated with translation efficiency and fine-tuning of translation [89–91]. In addition to synonymous codon usage in BVDV *Npro*, neighboring nucleotides flanking a codon seemed to mediate the selection of a specific codon from the synonymous codon group (Table 4 and Table S3). This strongly suggested that neighboring synonymous codon bias likely influences the formation of codon pair usage within the BVDV *Npro* region. Changes in codon pairs associated with CDCB may influence viral protein expression to some extent [34,64]. Furthermore, the codon pair context may represent an important factor in the phylogeny of individual species across Eukaryota, Archaea, and Bacteria [65,92]. Mutant viruses have the capacity to infect hosts that possess preexisting immune response from prior exposure to the virus. The higher the viral load present in the initial inoculum during transmission to a new host, the greater the likelihood of encountering a mutant virus capable of evading the existing immune response. Notably, a swarm of mutant viruses may exhibit a form of molecular memory, retaining genetic variants that conferred selective advantages during previous cycles of viral replication [93,94]. Based on *R* values (Table S3), the evolutionary patterns of the BVDV *Npro* region did not exhibit a genotype-specific model but revealed three distinct genetic groups (Figure 6). This intriguing evolutionary pattern may suggest three evolutionary pathways for the BVDV Npro coding sequence that are associated with its protein activity and function, directly benefiting viral reproduction and exaptation.

## Conclusion

Viral protein mutation plays an important role in the logic of virus evolution. BVDV Npro, serving as an important virulence factor for aiding virus replication, shows highly variable nucleotide usage patterns. We performed a comprehensive analysis of the evolutionary paradigms associated with variations in mononucleotide, nucleotide pair, synonymous codon, and codons with downstream neighboring nucleotides in the BVDV Npro coding sequence. Generally, both the overall nucleotide usage bias and synonymous codon usage bias are capable of displaying genotype-specific models to varying degrees. However, the extents of nucleotide pair usage and CDCB vary widely. Apart from the high mutation rate in the Npro coding sequence, the overall nucleotide usage, nucleotide pair, synonymous codon usage, and codons with downstream neighboring nucleotides can be strongly influenced by natural selective pressures derived from translational selection and the host’s immune response. Notably, both nucleotide pairs and CDCB in the BVDV Npro coding sequence exhibit a specific evolutionary paradigm distinct from genotype-specific patterns. The diverse repertoire of nucleotide pairs, synonymous codons, and CDCB in *Npro* could provide BVDV mutants with ample opportunities for direct appropriation as well as exaptation, allowing them to overcome the vast array of host defense systems. Taken together, the corresponding evolutionary paradigms regarding nucleotide usage in specific arrays may guide us in discovering new avenues for molecular diagnostics and the design of potential therapies.

## Supplementary Material

Table S1.doc

Table S3.xls

Table S2.doc

Figure S1.tif

Supplementary_Table_S3_Legend-_QVIR-2025-0017.R1_.doc

## Data Availability

The data that support the findings of this study are openly available in Science Data Bank at https://doi.org/10.57760/sciencedb.19428, reference number 95.
