# Supplementary material for: Mapping evolutionary paradigm of bovine viral diarrhea virus Npro associated with different organizations of nucleotide
Source: Virulence. 2025 Aug 29;16(1):2550620. doi: 10.1080/21505594.2025.2550620 (PMC12408059; doi:10.1080/21505594.2025.2550620)
Supplement: Table S1.doc [file KVIR_A_2550620_SM9381.doc]

**Table S1 The background information about BVDV strains selected in this study**

| Genbank accession number | Strain | Isolation | Time | Genotype |
| --- | --- | --- | --- | --- |
| KF501393.1 | BVDV JL-1 | China | 2009 | 1 |
| MH490943.1 | BVDV BJ-2016 | China | 2016 | 1 |
| MH490942.1 | BVDV BJ-2013 | China | 2013 | 1 |
| MF693403.1 | SWU-Z6 | China | 2016 | 1 |
| MN188074.1 | PI285 | USA | 2017 | 1 |
| MH166806.1 | XC | China | 2015 | 1 |
| MW732739.1 | YandaSpl | USA | 1993 | 1 |
| MW732738.1 | PI407 | USA | 2015 | 1 |
| MW713362.1 | PI819 | USA | 2017 | 1 |
| MW713361.1 | BoAEC1190 | USA | 2007 | 1 |
| MW655632.1 | SM09-20 | Switzerland | 2002 | 1 |
| MW655631.1 | S03-1175 | Switzerland | 2003 | 1 |
| MW655630.1 | R5013-96 | Switzerland | 1996 | 1 |
| MW655629.1 | R3572-90 | Switzerland | 1990 | 1 |
| MW655628.1 | R3230-95 | Switzerland | 1995 | 1 |
| MW655626.1 | Maria | Switzerland | 2004 | 1 |
| MW655625.1 | CH-04-01b | Switzerland | 2004 | 1 |
| MW250803.1 | BVDV 69-1 | UK | 2008 | 1 |
| MW054940.1 | MA/101/05 | Italy | 2005 | 1 |
| MW054939.1 | LO/151/09 | Italy | 2009 | 1 |
| MW054935.1 | TO/197/11 | Italy | 2011 | 1 |
| MW054934.1 | LA/87/05 | Italy | 2005 | 1 |
| MW054933.1 | LA/230/14 | Italy | 2014 | 1 |
| MW528233.1 | 2019BVD04889 | Germany | 2019 | 1 |
| MW528229.1 | 2018BVD06214 | Germany | 2018 | 1 |
| MW528226.1 | 2017BVD04597 | Germany | 2017 | 1 |
| MT977118.1 | BVDV 1b IT16/439 | Italy | 2016 | 1 |
| MK102095.1 | 20170226 | China | 2017 | 1 |
| KC695810.1 | camel-6 | China | 2010 | 1 |
| KF896608.1 | Bega-like | Australia | 2012 | 1 |
| KC695814.1 | Av69 VEDEVAC | USA | 2011 | 1 |
| MT654137.1 | 20-8536 | USA | 2020 | 1 |
| MW014288.1 | GXSS03 | China | 2018 | 1 |
| MN623291.1 | NX2019/01 | China | 2019 | 1 |
| MT079816.1 | GXNN1 | China | 2018 | 1 |
| MH379231.1 | P7A | USA | 2018 | 1 |
| MH379221.1 | P1 | USA | 2017 | 1 |
| KY849592.1 | SLO/2416/2002 | Slovenia | 2002 | 1 |
| MH899945.1 | SLO/1883/2013 | Slovenia | 2013 | 1 |
| MH899944.1 | SLO/28537/2017 | Slovenia | 2017 | 1 |
| MH899943.1 | SLO/1361/2014 | Slovenia | 2014 | 1 |
| MH899942.1 | SLO/33529/2015 | Slovenia | 2015 | 1 |
| MH231153.1 | Nebraska | USA | 1990 | 1 |
| NC_001461.1 | NADL | USA | 1988 | 1 |
| KX577637.1 | SLO/2407/2006 | Slovenia | 2006 | 1 |
| MH379638.1 | Ho916 | UK | 1993 | 1 |
| MF172980.1 | GSTZ | China | 2012 | 1 |
| MF278652.1 | XZ02 | China | 2016 | 1 |
| KX857724.1 | ACM/BR/2016 | Brazil | 2016 | 1 |
| LT631725.1 | UM/126/07 | Italy | 2007 | 1 |
| KT943518.1 | BJ120 | China | 2012 | 1 |
| KU159365.1 | USII-S15 | USA | 2015 | 1 |
| KR029825.1 | Egy/Ismailia/2014 | Egypt | 2014 | 1 |
| LC089876.1 | Shitara/02/06 | Japan | 2006 | 1 |
| LC089875.1 | IS26/01ncp | Japan | 2001 | 1 |
| KR866116.1 | SD-15 | China | 2015 | 1 |
| KP313732.1 | Carlito | Switzerland | 2014 | 1 |
| KP941591.1 | USMARC-55925 | USA | 2014 | 1 |
| JN400273.1 | SD0803 | China | 2008 | 1 |
| KJ689448.1 | GX4 | China | 2012 | 1 |
| KJ541471.1 | GS5 | China | 2013 | 1 |
| KC963967.1 | 12F004 | South Korea | 2012 | 1 |
| KC757383.1 | 10JJ-SKR | South Korea | 2010 | 1 |
| KC853441.1 | SuwaCp | Switzerland | 1993 | 1 |
| KC853440.1 | SuwaNcp | Switzerland | 1993 | 1 |
| JX419398.1 | RK13/END+ | Japan | 2008 | 1 |
| JX419397.1 | RK13/END- strain | Japan | 2008 | 1 |
| JQ799141.1 | M31182 | China | 2010 | 1 |
| KF772785.1 | CC13B | China | 2013 | 1 |
| NC_039237.1 | 890 | USA | 1992 | 2 |
| JF714967.1 | HLJ-10 | China | 2011 | 2 |
| FJ527854.1 | XJ-04 | China | 2004 | 2 |
| MW528235.1 | TV02-13_T136 | Germany | 2013 | 2 |
| MW528234.1 | D66-11-28 | Germany | 2011 | 2 |
| GQ888686.1 | JZ05-1 | China | 2005 | 2 |
| FJ040215.1 | Th/04_KhonKaen | Thailand | 2004 | 3 |
| KY683847.1 | SV757/15 | Brazil | 2015 | 3 |
| MH410812.1 | LV168-36/16RN | Brazil | 2013 | 3 |
| MH410816.1 | LV03/12 | Brazil | 2011 | 3 |
| JQ612704.1 | Italy-83/10-ncp | Italy | 2010 | 3 |
| JX469119.1 | JS12/01 | China | 2012 | 3 |
| MH410815.1 | LV127-29/16MA | Brazil | 2013 | 3 |
| KJ627179.1 | Italy-68/13ncp | Italy | 2013 | 3 |
| KY762287.2 | PB22487 | Brazil | 2012 | 3 |
| MH410814.1 | LV125-8/16MA | Brazil | 2013 | 3 |
| KC297709.1 | LVRI/cont-1 | South America | 2013 | 3 |
| KC788748.1 | Italy-129/07 | Italy | 2007 | 3 |
| KY767958.1 | SV478/07 | Brazil | 2007 | 3 |
| HQ231763.1 | Italy-1/10-1 | Italy | 2010 | 3 |
| KU563155.1 | HN1507 | China | 2015 | 3 |
| KJ627180.1 | Italy-68/13cp | Italy | 2013 | 3 |
| JQ612705.1 | Italy-83/10-cp | Italy | 2010 | 3 |
| KP941585.1 | USMARC-55476 | USA | 2014 | 2 |
| MH231138.1 | MnFetus | USA | 1991 | 2 |
| KC963968.1 | 11F011 | South Korea | 2011 | 2 |
