# Supplementary material for: Mapping evolutionary paradigm of bovine viral diarrhea virus Npro associated with different organizations of nucleotide
Source: Virulence. 2025 Aug 29;16(1):2550620. doi: 10.1080/21505594.2025.2550620 (PMC12408059; doi:10.1080/21505594.2025.2550620)
Supplement: Table S2.doc [file KVIR_A_2550620_SM9379.doc]

**Table S2 Nucleotide composition variations in BVDV Npro coding sequence**

| Nucleotide composition | Genotype 1 | Genotype 2 | Genotype 3 |
| --- | --- | --- | --- |
| ^a^U% | 22.134±1.091 | 21.602±0.524 | 22.370±0.471 |
| ^a^C% | 20.227±0.907 | 22.222±0.636 | 21.025±0.591 |
| ^a^A% | 32.290±0.910 | 30.928±0.707 | 29.680±0.399 |
| ^a^G% | 25.349±1.026 | 25.248±0.549 | 26.924±0.415 |
| ^b^U1% | 19.436±1.432 | 18.824±0.443 | 18.552±0.586 |
| ^b^C1% | 19.712±1.398 | 20.685±0.616 | 21.131+0.586 |
| ^b^A1% | 31.573±1.293 | 31.622±0.742 | 27.943±1.199 |
| ^b^G1% | 29.279±1.027 | 28.869±0.955 | 32.374±1.227 |
| ^c^U2% | 24.888±0.812 | 24.702±0.318 | 26.753±0.749 |
| ^c^C2% | 17.969±0.959 | 18.229±0.308 | 17.791±0.495 |
| ^c^A2% | 33.385±1.194 | 31.250±0.779 | 33.135±0.842 |
| ^c^G2% | 23.758±1.353 | 25.818±0.707 | 22.321±1.066 |
| ^d^U3% | 22.078±2.877 | 21.280±1.615 | 21.807±0.840 |
| ^d^C3% | 23.000±2.344 | 27.753±1.741 | 24.156±1.358 |
| ^d^A3% | 31.913±1.800 | 29.911±1.449 | 27.961±0.897 |
| ^d^G3% | 23.009±1.960 | 21.057±1.524 | 26.076±1.291 |

^a^stands for mononucleotide composition variant composed of the mean value ± standard error in the whole Npro coding sequence.

^b^means mononucleotide composition variant composed of the mean value ± standard error in the first codon position of Npro coding sequence.

^c^denotes mononucleotide composition variant composed of the mean value ± standard error in the second codon position of Npro coding sequence.

^d^means mononucleotide composition variant composed of the mean value ± standard error in the third codon position of Npro coding sequence.
