# Supplementary figures and images for: Mapping evolutionary paradigm of bovine viral diarrhea virus Npro associated with different organizations of nucleotide
Source: Virulence. 2025 Aug 29;16(1):2550620. doi: 10.1080/21505594.2025.2550620 (PMC12408059; doi:10.1080/21505594.2025.2550620)

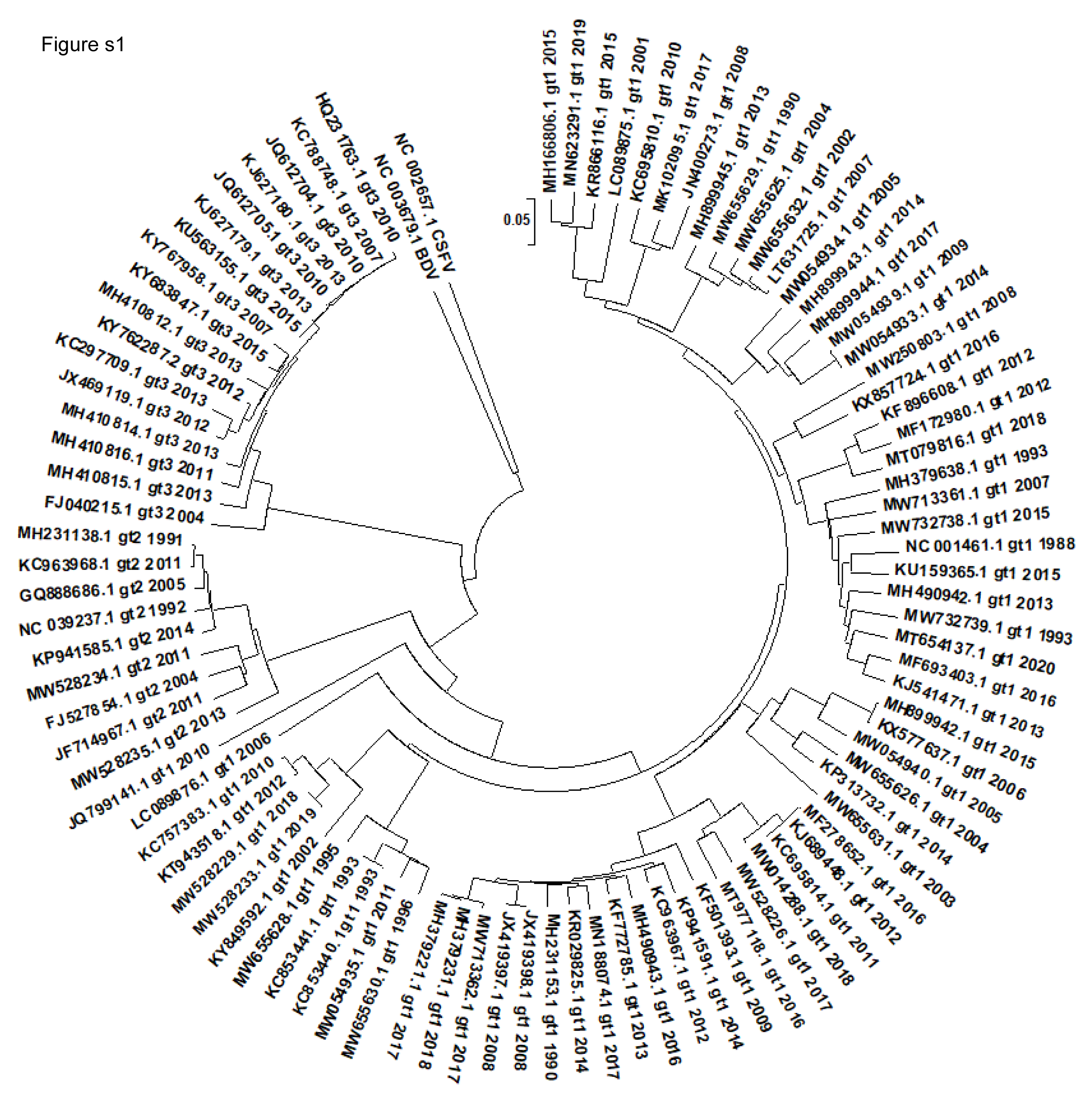

Supplement: Figure S1.tif [file KVIR_A_2550620_SM9378.tif]
