# Supplementary material for: Mapping evolutionary paradigm of bovine viral diarrhea virus Npro associated with different organizations of nucleotide
Source: Virulence. 2025 Aug 29;16(1):2550620. doi: 10.1080/21505594.2025.2550620 (PMC12408059; doi:10.1080/21505594.2025.2550620)
Supplement: Supplementary_Table_S3_Legend-_QVIR-2025-0017.R1_.doc [file KVIR_A_2550620_SM9377.doc]

Table S3 The R data for Npro coding sequence of BVDV
